# COPAR: A ChIP-Seq Optimal Peak Analyzer

**DOI:** 10.1155/2017/5346793

**Published:** 2017-03-05

**Authors:** Binhua Tang, Xihan Wang, Victor X. Jin

**Affiliations:** ^1^Epigenetics & Function Group, School of Internet of Things, Hohai University, Jiangsu 213022, China; ^2^School of Public Health & Biostatistics, Shanghai Jiao Tong University, Shanghai 200025, China; ^3^Department of Molecular Medicine & Biostatistics, University of Texas Health Science Center, San Antonio, TX 78249, USA

## Abstract

Sequencing data quality and peak alignment efficiency of ChIP-sequencing profiles are directly related to the reliability and reproducibility of NGS experiments. Till now, there is no tool specifically designed for optimal peak alignment estimation and quality-related genomic feature extraction for ChIP-sequencing profiles. We developed open-sourced COPAR, a user-friendly package, to statistically investigate, quantify, and visualize the optimal peak alignment and inherent genomic features using ChIP-seq data from NGS experiments. It provides a versatile perspective for biologists to perform quality-check for high-throughput experiments and optimize their experiment design. The package COPAR can process mapped ChIP-seq read file in BED format and output statistically sound results for multiple high-throughput experiments. Together with three public ChIP-seq data sets verified with the developed package, we have deposited COPAR on GitHub under a GNU GPL license.

## 1. Introduction

Next-generation sequencing (NGS) integrated with ChIP technology provides a genome-wide perspective for biomedical research and clinical diagnosis applications [1–3].

Data quality and peak alignment of ChIP-sequencing profiles are directly related to the reliability and reproducibility of analysis results. For example, ChIP-seq data characterize alteration evidence for transcription factor (TF) binding activities in response to chemical or environmental stimuli, but if the ChIP-seq alignment is poorly selected, any follow-up analysis may lead to inaccurate TF binding results and inevitable loss of biological meanings [4, 5].

The mostly investigated items in ChIP-seq peak calling procedures are peak number, false discovery rate (FDR), corresponding bin-size, and other statistical thresholds selected in each analysis. Without exception, such arguments form impenetrable barriers for biologists and bioinformaticians to choose a suitable pair condition for analyzing experimental results.

And to our knowledge, few literatures or application notes focus on such topics; thus herein we propose a flexible package based on feature extraction and signal processing algorithms for solving such an argument-selection optimization problem in optimal peak alignment.

In summary, the package COPAR can quantitatively measure NGS/ChIP-seq experiment quality through global peak alignment comparison and extract genomic features based on spectrum method for in-depth analysis of ChIP-sequencing profiles.

## 2. Materials and Methods

### 2.1. Optimal Peak Alignment Estimation

For determining optimal ChIP-seq alignment, we need to analyze peak numbers under specific argument constraints. Thus we acquire optimal peak numbers by constraining specific arguments, which can be formalized as a class of optimal track analysis, illustrated as

(1) arg maxi Pi,i∈Ns.t. fi≤χ, bi=β, pi≤δ,

where *P*_*i*_ denotes a set of optimal peak numbers under corresponding argument constraints, *f*_*i*_ stands for argument FDR, *b*_*i*_ stands for bin-size, *p*_*i*_ denotes *p* value threshold, and *χ*, *β*, and *δ* represent the presupposed argument values, respectively.

### 2.2. Spectrum-Based Genomic Feature Extraction

For a finite random variable sequence, its power spectrum is normally estimated from its autocorrelation sequence by use of discrete-time Fourier transform (DTFT), denoted as [6–8]

(2) $$\begin{array}{c} P\left(\;\omega \;\right)=\frac{1}{2\pi }\overset{\infty }{\underset{n=-\infty }{\sum }}C_{xx}\left(\;n\;\right)e^{-jn\omega }, \end{array}$$

where *C*_*xx*_ denotes autocorrelation sequence of a discrete signal *x*_*n*_, defined as

(3) $$\begin{array}{c} C_{xx}\left(\;i,j\;\right)=\frac{E\left[\left(X_{i}-\mu_{i}\right)\left(X_{j}-\mu_{j}\right)\right]}{\sigma_{i}\sigma_{j}}, \end{array}$$

where *μ* and *σ* stand for mean and variance, respectively.

In our study, for consideration of the ChIP-seq data characteristics, we use 128 sampling points to calculate discrete Fourier transform, with the related sampling frequency 1 KHz.

## 3. Results

The COPAR package was developed and open-sourced for academic biologists, and it uses built-in functions for determining optimal peak alignment candidate and extracting genomic features from ChIP-seq dataset.

**Figure 1:**
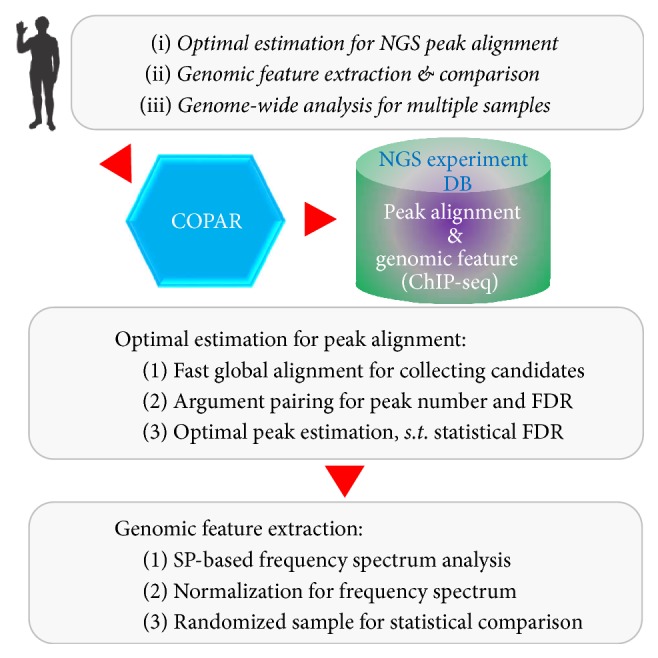
Flowchart for optimal peak alignment estimation and genomic feature analysis with COPAR. The package can perform optimal peak estimation based on global alignment of ChIP-seq data; then it can utilize the frequency spectrum approach for genomic feature extraction and carries out statistical comparison for multiple ChIP-seq samples.

The package is designed to handle BED-formatted ChIP-seq data as input [9], and it can process single ChIP-seq for optimal peak alignment and feature extraction analysis, together with the capability to perform genome-wide statistical comparison for multiple ChIP-seq samples. The analysis flowchart for the package is given in Figure 1.

It can automatically determine the optimal peak alignment with statistically meaningful FDR through fast global alignment comparison; the global comparison is subject to two statistical arguments, namely, bin-size and *p* value threshold.

**Figure 2:**
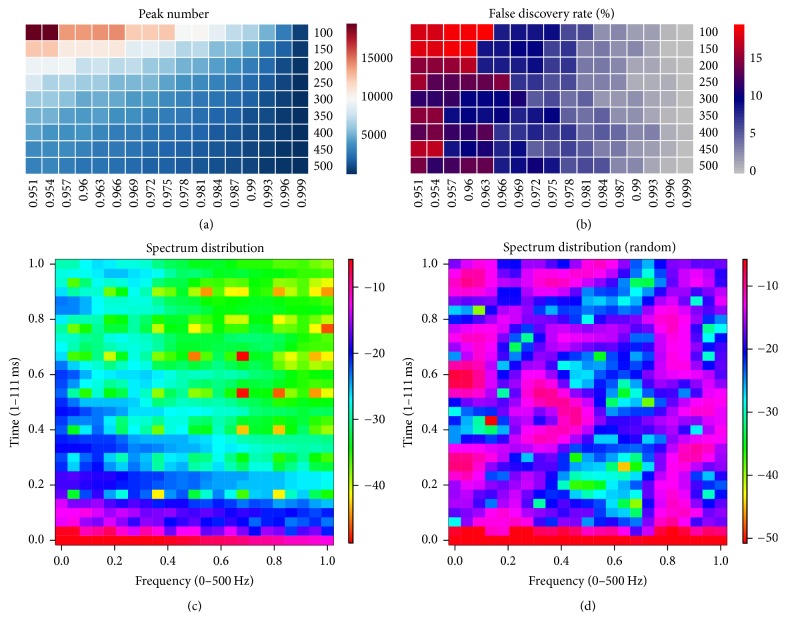
*Global optimal peak analysis result subject to the arguments bin-size and FDR*. (a) Global distributions for peak number candidates and (b) corresponding false discovery rate, subject to bin-size (vertical axis, from 100 through 500 bp) and *p* value threshold (horizontal axis, from 0.951 to 0.999), respectively; (c) genomic feature extraction based on spectrum distribution for global peak number candidates identified from COPAR; (d) spectrum distribution for the randomized sequence.

The functionalities of our developed package are largely complementary to and extend current tools used for ChIP-seq data analysis. The optimal peak alignment estimation is shown in Figures 2(a) and 2(b); and the spectrum-based feature extraction is given in Figures 2(c) and 2(d). Figures 2(a) and 2(b) utilize heatmap to represent peak number and corresponding FDR candidate subject to each argument pair, bin-size (vertical axis), and *p* value threshold (horizontal axis), respectively; Figure 2(c) denotes the spectrum distribution of the global peak alignment candidate sequence, normalized with its frequency range [0,500] Hz and magnitude within [−40, −3] dB; Figure 2(d) denotes the randomized case.

## 4. Conclusions

Based on global peak alignment, COPAR optimizes the argument selection in ChIP-seq analysis; meanwhile, COPAR utilizes the signal spectrum processing method to further extract genomic features and statistically compare multiple ChIP-seq samples for NGS high-throughput experiments.

In summary, our developed package COPAR can process mapped read file in BED format and output statistically sound results for diverse high-throughput sequencing experiments; we further verified the package with three GEO ChIP-seq datasets as study cases, and we included the analysis results into the package manual. The developed package COPAR is currently available under a GNU GPL license from https://github.com/gladex/COPAR.

## Abbreviations

NGS: Next-generation sequencing
ChIP-seq: Chromatin immunoprecipitation-sequencing
FDR: False discovery rate
TF: Transcription factor
DTFT: Discrete-time Fourier transform.
